# Editorial for Special Issue: Achilles Curse and Remedy: Tendon Diseases from Pathophysiology to Novel Therapeutic Approaches

**DOI:** 10.3390/ijms21207454

**Published:** 2020-10-09

**Authors:** Denitsa Docheva

**Affiliations:** Experimental Trauma Surgery, Department of Trauma Surgery, University Medical Center Regensburg, Am Biopark 9, 93053 Regensburg, Germany; denitsa.docheva@ukr.de; Tel.: +49-941-943-1605

In Greek mythology, Achilles, the Greek hero, is almost invulnerable—except for his Achilles heel, whose injury resulted in his death. How could a tendon injury take such a prominent place in Greek mythology? This injury was obviously such a crucial and inexplicable event that it was extensively honored in the legendary Iliad of Homer. Presumably, the ancient Greeks had already asked themselves how it could have happened that the greatest tendon of man could suddenly break, even in a young, vigorous athlete.

Tendons are dense connective tissues and critical components for the integrity and function of the musculoskeletal system, as they connect bone to muscle and transmit forces on which locomotion entirely depends. Due to the increasing age of our society and a rise in the engagement of young people in overuse activities or extreme sports, tendon diseases present major clinical and financial challenges in modern medicine. Inevitably, tendinopathies lead to the final stage disease that is tendon rupture, and once this happens, tendon natural healing is slow, often poorly responding to treatments and requiring prolonged rehabilitation in most cases. A major cause of tendon rupture is tendon tissue degeneration, a process that can be considered a failure of matrix adaptation and remodeling because of an imbalance between matrix synthesis and break down due to a variety of stresses and mechanical loads.

There are three main hypotheses about the cause(s) of tendon degeneration: (1) mechanical overuse (via matrix), (2) neo-vascularization (via exogenous cells), and (3) cell and tissue aging (via endogenous cells). Most likely, these three all trigger cross-talk and cross-react with one another, ultimately leading to the failure of the whole tendon unit. To date, there have been only a few approved treatments for tendinopathy that are targeted against specific molecular processes, and still, in most cases, there is little to no evidence of therapeutic effectiveness, especially in the long term. Concerning the therapy of the end-stage disease that is tendon rupture, there are two main clinical algorithms, namely, subjecting patients to surgical or conservative therapy, and both require months-long periods to achieve mostly partial and rarely full structural and functional tendon reconstitution.

This Special Issue aimed to embrace research and review articles concentrating on:

1. The spectrum of tendon pathologies: e.g., triggers, trails, and end-state of tendinopathy by Steinmann et al. [1].
2. Endogenous tendon cells and their governing molecular pathways: e.g., cell–extracellular matrix (ECM) contacts mediated via α2β1 integrin by Kronenberg et al. [2]; transforming growth factor beta-activated kinase-1 (TAK 1) signaling in tendon and enthesis by Friese et al. [3]; and transcriptional activity of the early growth response 1 (*EGR1*) gene in healthy and scarred tendons by Havis et al. [4].
3. Cross-talk with exogenous cells: e.g., with macrophages during inflammation by Vinhas et al. [5] and myoblasts at the site of myotendinous junction by Strenzke et al. [6].
4. Niche chemical composition: e.g., uremic toxins and antibiotics in pathological kidney conditions by Popowski et al. [7]; tissue chemical changes during tendon aging by Yin et al. [8]; and cartilage oligomeric matrix protein (COMP) fragmentation in tendon injury by Smith et al. [9].
5. Tendon structural composition and biomechanical properties: e.g., alterations in elastic properties after tendon injury by Frankewycz et al. [10] and in vitro and in vivo response of tenocytes to mechanical stimulation by Fleischhacker et al. [11].
6. Medicinal and tissue engineering therapeutic approaches: e.g., pulsed electromagnetic field (PEMF)-based therapy by Vinhas et al. [5]; various percutaneous treatments by Darrieutort-Laffite et al. [12]; enthesial regenerative approaches by Friese et al. [3]; and current tendinopathy management strategies by Steinmann et al. [1].

A total of eight original articles and four review articles are published, as summarized in Table 1.

## Figures and Tables

**Table 1:** Summary of the articles published in the Special Issue [1,2,3,4,5,6,7,8,9,10,11,12].

Article Type	Authors	Main Message
Original Research Articles	Adriana Vinhas, Ana F. Almeida, Ana I. Gonçalves, Márcia T. Rodrigues, and Manuela E. Gomes [5]	Cross-talk between macrophages and human tendon-derived cells (hTDCs), using co-culture model with and without IL-1β stimulation, was investigated. Furthermore, the potential modulatory effect of pulsed electromagnetic fields (PEMFs) was examined. The PEMFs influenced a macrophage pro-regenerative and anti-inflammatory phenotype, and overcame the effect of IL-1β-treated hTDCs, thus suggesting a beneficial role of the PEMF in guiding inflammatory responses toward tissue regeneration.
		In vitro studies
	Erman Popowski, Benjamin Kohl, Tobias Schneider, Joachim Jankowski, and Gundula Schulze-Tanzil [7]	The effects of the uremic toxins phenylacetic acid (PAA) and quinolinic acid (QA), both alone and in combination with ciprofloxacin (CPX), on human tenocytes were studied. CPX, administered at a therapeutic concentration, suppressed tenocyte metabolism. Combinations of CPX with PAA or QA did not cause greater cytotoxicity than incubation with CPX alone. Gene expression of matrix metalloproteinase *MMP-1* was increased, while the pro-inflammatory cytokine *IL-1β* was reduced by CPX, but up-regulated by PAA and QA. Type I collagen protein decreased only in response to high CPX doses, demonstrating that CPX was more tenotoxic than the uremic toxins.
		In vitro studies
	Borys Frankewycz, Leopold Henssler, Johannes Weber, Natascha Platz Batista da Silva, Matthias Koch, Ernst Michael Jung, Denitsa Docheva, Volker Alt, and Christian G. Pfeifer [10]	Using shear wave elastography technology, 12 patients who suffered from an acute Achilles tendon rupture were acquired and monitored for one year, revealing a significant increase in tendon scar elastic properties within the first six weeks and a second significant increase three to six months after injury. This pilot study suggested a time correlation of biomechanical properties with the biological healing phases of tendon tissue, which could be implemented in treatment and aftercare protocols.
		Patient study
	Daniel Kronenberg, Philipp A. Michel, Eva Hochstrat, Ma Wei, Jürgen Brinckmann, Marcus Müller, Andre Frank, Uwe Hansen, Beate Eckes and Richard Stange [2]	Tendon tissue and cells from wild-type and integrin α2β1 knockout mice were compared. Significantly smaller collagen fibrils, altered dynamic E-modulus, increased C-terminal fragments of type I collagen, and MMP-2 activity were detected in α2β1-deficient tendons. Moreover, the mutant tenocytes produced more collagen in vitro. These results report a significant role of α2β1 in tendon tissue and a potential link to chronic tendinosis.
		In vivo and in vitro studies
	Roger Smith, Patrik Önnerfjord, Kristin Holmgren, Shacko di Grado, and Jayesh Dudhia [9]	Cartilage oligomeric matrix protein (COMP) fragments were purified from synovial fluids of horses with intra-thecal tendon injuries and media from equine tendon explants, and analyzed by mass spectrometry and competitive inhibition ELISA, revealing a significant increase in the COMP neo-epitope with tendon injury, suggesting that this assay could be suitable for clinical use.
		In vivo and in vitro studies
	Nai-Hao Yin, Anthony W. Parker, Pavel Matousek, and Helen L. Birch [8]	In order to gain better understanding of the chemical differences between young and old tendons, fluorescence and Raman combined spectra analyses were carried out with vacuum-dried young and old equine superficial digital flexor and deep digital flexor tendons. The analysis indicated increased fluorescence, changes in intra-tendinous fluorophores, a decline in cellular numbers, and an accumulation of advanced glycation end products as tendons aged, signifying that Raman spectroscopy can successfully detect age-related tendon molecular differences.
		In vivo studies
	Maximilian Strenzke, Paolo Alberton, Attila Aszodi, Denitsa Docheva, Elisabeth Haas, Christian Kammerlander, Wolfgang Böcker, and Maximilian Michael Saller [6]	To investigate new mechanisms in musculotendinous interaction, myoblasts were exposed to a secretome obtained from a Scleraxis-overexpressing mesenchymal stem cell (MSC) line, which led to a significant increase in myoblast fusion and metabolic activity, but had no effect on myoblast migration and myofiber alignment. RNA sequencing revealed differential expression of genes encoding ECM proteins, transmembrane receptors and proteases between native MSCs and Scleraxis MSCs.
		In vitro studies
	Viviane Fleischhacker, Franka Klatte-Schulz, Susann Minkwitz, Aysha Schmock, Maximilian Rummler, Anne Seliger, Bettina M. Willie, and Britt Wildemann [11]	Comparative analysis of in vitro and in vivo cyclic compressive loading of Achilles tendon-derived tenocytes revealed similar expression profiles of tendon-associated gene markers, but interestingly, significant differences in cell shape and gene expression levels of *collagen I*, *collagen III*, and *MMPs.* These data can help in understanding how closely in vitro stimulation of tenocytes mimics the in vivo situation.
		In vivo and in vitro studies
Review Articles	Christelle Darrieutort-Laffite, Louis J. Soslowsky, and Benoit Le Goff [12]	Various percutaneous treatments have been applied to tendon lesions: e.g., injectable treatments, platelet-rich plasma, corticosteroids, stem cells, MMP inhibitors, and anti-angiogenic agents. This review summarized the molecular and structural effects of such treatments obtained in vitro and in vivo, as well as their efficacy in clinical trials, and concluded that local treatments had some impact on neovascularization, inflammation, or tissue remodeling in animal models, but clinical evidence remained too weak, therefore, further studies are needed to evaluate their value.
	Nina Friese, Mattis Benno Gierschner, Patrik Schadzek, Yvonne Roger, and Andrea Hoffmann [3]	This review focused on the transition zone from bone to tendon—the enthesis, its anatomical structure, development during embryogenesis, and dysfunction during inflammation, with particular attention on the role of a signaling mediator protein, transforming growth factor beta-activated kinase-1 (TAK1). Furthermore, a short synopsis on the current progress in restorative strategies for damaged enthesis was given.
	Emmanuelle Havis and Delphine Duprez [4]	This review provided a broad overview on the roles of the early growth response 1 (*EGR1*) gene in tendon, cartilage, bone, and adipose tissues, as well as on EGR1 involvement in tissue and organ fibrosis and on the link between its transcriptional activity and abnormal ECM production, suggesting EGR1 as a potential therapeutic target to fight fibrotic conditions or to modulate tendon repair.
	Sara Steinmann, Christian G. Pfeifer, Christoph Brochhausen, and Denitsa Docheva [1]	This review encompassed detailed information on morbidity and clinical relevance of tendinopathies; histopathological, structural, cellular, epigenetic, transcriptomic, proteomic, and metabolomic changes during tendinopathy; involvement of vasculature, inflammation, and neurons; and biochemical and biomechanical alterations, as well as on the current tendinopathy management strategies. A graphical summary on the current knowledge of triggers, trails, and the end state of tendinopathies was provided. The review anticipated a more systematic and multidisciplinary approach to decipher the complexity behind tendinopathy and to outline possible ways forward to combat tendinopathy.
